# Supplementary material for: Identification of photoperiod-induced specific miRNAs in the adrenal glands of Sunite sheep (Ovis aries)
Source: Front Vet Sci. 2022 Jul 22;9:888207. doi: 10.3389/fvets.2022.888207 (PMC9354845; doi:10.3389/fvets.2022.888207)
Supplement: Supplementary file 1 [file Data_Sheet_1.ZIP › Supplementary file/miRDeep2 file.zip!/miRDeep2 file/Know_Structure/SP42A3/oar-mir-374b.pdf]

miRBase precursor : oar-mir-374b
Total read count : 24328
oar-miR-374b read count : 23817
remaining reads : 511

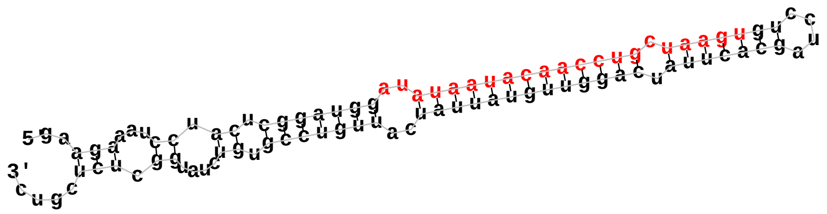

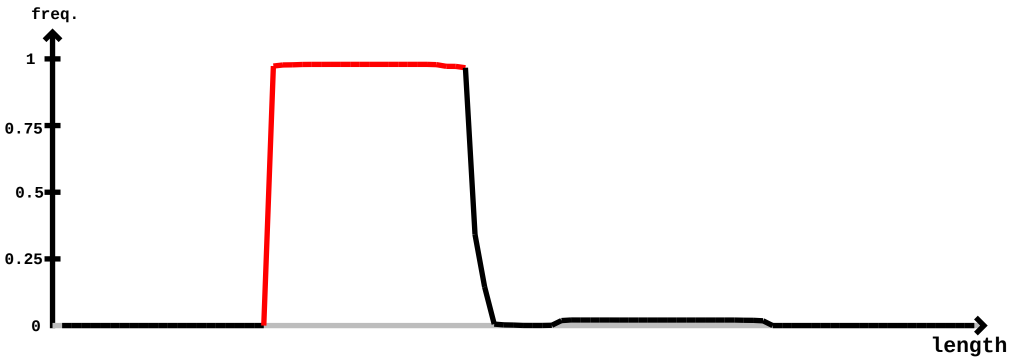


```
                                         oar-miR-374b
5'- gaagaaauccuacucggauggauauaaauacaaccugcuaaguguccuagcacuuaucagguuguauuaucauuguccgugucuauggcucucguc  -3'    exp
    ((((..((...((.(((((((..((((((((((((.(((((((....))))))).))))))))))))..))))))).))..))..)).))...     reads   mm   sample
    ....................gauauaaauacaaccugcuaagu.....................................................   1       0    seq
    .....................Cuauaaauacaaccugcu.........................................................   1       1    seq
    .....................auauaaauacaaccugAu.........................................................   1       1    seq
    .....................auauGauacaaccugcu..........................................................   1       1    seq
    .....................auauaaauacaaccugcu.........................................................   22      0    seq
    .....................auauaaauacaaccugcua........................................................   152     0    seq
    .....................auauaaauacaaccugcuaa.......................................................   7       0    seq
    .....................auauaaauacaaccugcuaag......................................................   125     0    seq
    .....................auauaaauacaaccugcuaagu.....................................................   15203   0    seq
    .....................auauaaauacaaccugcuaagug....................................................   4768    0    seq
    .....................auauaaauacaaccugcuaagugu...................................................   3318    0    seq
    .....................auauaaauacaaccugcuaaguguc..................................................   54      0    seq
    .....................auauaaauacaaccugcuaagugucc.................................................   9       0    seq
    .....................auauaaauacaaccugcuaaguguccu................................................   6       0    seq
    .....................auauaaauacaaccugcuaaguguccuag..............................................   1       0    seq
    .....................auauaaauacaaccugcuaaguguccuagca............................................   1       0    seq
    .....................auauaaauacaaccugcuaaguguccuagcacu..........................................   2       0    seq
    .....................auauaaauacaaccugcuaaguguccuagcacuuauc......................................   1       0    seq
    ......................uauaaauacaaccugcuaagu.....................................................   12      0    seq
    ......................uauaaauacaaccugcuaagug....................................................   4       0    seq
    ......................uauaaauacaaccugcuaagugu...................................................   70      0    seq
    ......................uauaaauacaaccugcuaaguguc..................................................   12      0    seq
    ......................uauaaauacaaccugcuaagugucc.................................................   2       0    seq
    ......................uauaaauacaaccugcuaaguguccu................................................   1       0    seq
    .......................auaaauacaaccugcuaagu.....................................................   2       0    seq
    .......................auaaauacaaccugcuaagugu...................................................   1       0    seq
    .......................auaaauacaaccugcuaaguguc..................................................   2       0    seq
    .......................auaaauacaaccugcuaagugucc.................................................   3       0    seq
    .......................auaaauacaaccugcuaaguguccu................................................   3       0    seq
    ........................uaauacaaccugcuaagugA....................................................   2       1    seq
    ........................uaauacaaccugcuaagugu....................................................   3       0    seq
    ........................uaauacaaccugcuaaguguc...................................................   1       0    seq
    ........................uaauacaaccugcuaagugucc..................................................   4       0    seq
    ........................uaauacaaccugcuaaguguccu.................................................   21      0    seq
    ........................uaauacaaccugcuaaguguccua................................................   1       0    seq
```

oar-miR-374b

gaagaaauccuacucggauggauauaauacaaccugcuaaguguccuagcacuuaucagguuguauuaucauuguccgugucuauggcucucguc			
.........................aauacaaccugcuaaguguccu..	5	0	seq
.........................aauacaaccugcuaaguguccuag..	1	0	seq
.........................aauacaaccugcuaaguguccuagc...	1	0	seq
..........................auacaaccugcuaaguguccu..	1	0	seq
...........................uacaaccugcuaaguguccuagc...	1	0	seq
....cacuuaucagguuguauuauc.........................	1	0	seq
....acuuaucagguuguauuauc.........................	8	0	seq
....acuuaucagguuguauuauA.........................	1	1	seq
....acuuaucagguuguauuauca........................	2	0	seq
....acuuaucagguuguauuaucau.......................	10	0	seq
....acuuaucagguuguauuaucauu......................	1	0	seq
....cuuaucagguuguauuauA.........................	2	1	seq
....cuuaucagguuguauuauc.........................	6	0	seq
....cuuaucagguuguauuaucU........................	2	1	seq
....cuuaucagguuguauuauca........................	3	0	seq
....cuuaucagguuguauuaucau.......................	33	0	seq
....cuuaucagguuguauuaucauu......................	383	0	seq
....uuaucagguuguauuaucau.......................	2	0	seq
....uuaucagguuguauuaucauu......................	47	0	seq
....uuaucagguuguauuaucauug.....................	1	0	seq
